# Associations of Leisure-Time Internet and Computer Use With Overweight and Obesity, Physical Activity and Sedentary Behaviors: Cross-Sectional Study

**DOI:** 10.2196/jmir.1084

**Published:** 2009-07-27

**Authors:** Corneel Vandelanotte, Takemi Sugiyama, Paul Gardiner, Neville Owen

**Affiliations:** ^2^Cancer Prevention Research CentreUniversity of QueenslandHerstonAustralia; ^1^Institute for Health and Social Sciences ResearchCentral Queensland UniversityRockhamptonAustralia

**Keywords:** Internet, computer, overweight, obese, BMI, body mass index, physical activity, human activities, sedentary behavior, leisure-time, leisure activities

## Abstract

**Background:**

Internet and computer use are increasingly common leisure-time sedentary behaviors, which have the potential to impact negatively on health outcomes. However, little is known about the extent to which adults’ Internet and computer use is associated with weight status and time spent in leisure-time physical activity.

**Objective:**

The objective is to examine associations of leisure-time Internet and computer use with overweight and obesity, leisure-time physical activity, and other sedentary behaviors.

**Methods:**

Participants (2650 adults living in Adelaide, Australia) completed a mail-back questionnaire including items on their height and weight, past seven day recall of leisure-time physical activity, Internet and computer use, and other leisure-time sedentary behaviors. Leisure-time Internet and computer use was categorized into no use, low use (less than three hours per week), or high use (three hours or more per week).

**Results:**

Participants with low leisure-time Internet and computer use had the highest levels of educational attainment and employment, and engaged in less other sedentary behaviors when compared to participants with no or high Internet and computer use. Multinomial logistic regression, adjusted for gender, age, employment, education, other sedentary behaviors and physical activity, determined that participants with a high leisure-time Internet and computer use were 1.46 (95% CI = 1.10 - 1.93) times more likely to be overweight (BMI≥25 and < 30 kg/m^2^) and 2.52 times more likely (95% CI = 1.82 - 3.52) to be obese (BMI≥30 kg/m^2^), compared to those who reported no Internet and computer use in their leisure-time. Adults with high leisure-time Internet and computer use were more likely to be overweight or obese even if they were highly active in their leisure time (OR = 1.86; 95% CI = 1.21 - 2.88), as compared to participants who did not use the Internet or computer. Leisure-time physical activity levels were largely independent of Internet and computer use.

**Conclusion:**

These findings suggest that, apart from nutritional and physical activity interventions, it may also be necessary to decrease time spent in sedentary behaviors, such as leisure-time Internet and computer use, in order to reduce the prevalence of overweight and obesity. Future Internet interventions to reduce weight or increase physical activity may need to differentiate between participants with different levels of Internet use in order to increase their effectiveness. Longitudinal studies are required to examine further the potential causal relationships between the development of overweight and specific sedentary behaviors such as Internet and computer use.

## Introduction

Many studies have shown that physical inactivity is associated with higher levels of overweight and obesity and that physical activity is essential in the prevention and treatment of overweight and obesity [1,2]. Recently, this evidence has led to the development of specific physical activity guidelines for overweight and obese people [3,4] which state that 60 to 90 minutes of daily moderate to vigorous physical activity are necessary to lose weight or to maintain weight loss.

There are strong adverse associations between time spent in sedentary behaviors and different health indicators [5-7], including the increased likelihood of being overweight or obese [6,8-10]. It is now generally accepted that “sedentariness” and physical (in)activity are two distinct classes of behavior, each with their own determinants [11]. They have independent effects on total energy expenditure, weight, and metabolic variables [10].

However, most of the evidence on associations between sedentary behavior and health outcomes, such as weight status and levels of physical activity, is specific to time spent watching television [5,6,8,10,12], which is the most commonly studied leisure-time sedentary behavior. Associations of health outcomes with other sedentary behaviors such as Internet and computer use remain largely unknown. In Australia, more than 70% of the population has access to the Internet, and this number is still increasing [13]. Internet and computer use are increasingly common leisure-time sedentary behaviors [13,14] which have the potential to impact negatively on health, independent of other sedentary behaviors. Extensive use of the Internet and computers may also displace time spent in leisure-time physical activity.

Several studies have examined the associations between leisure-time Internet and computer use, physical activity, and levels of overweight/obesity in children and adolescents, with inconsistent outcomes. Some studies show that high leisure-time Internet and computer use is associated with higher Body Mass Index (BMI) and lower physical activity levels [15-18]; other studies are not able to confirm this [19-21]. However, to our knowledge no studies have evaluated these relationships in adults.

Further, little is known about how sedentary behaviors relate to each other. In relation to health outcomes, it is important to know whether high leisure-time Internet and computer use is a marker for high levels of other sedentary behaviors. It may be that leisure-time Internet and computer use is related to poor health outcomes due to its association with a broader pattern of sedentary behavior. A study by Sugiyama et al [22] demonstrated that, in women, time spent watching TV was associated positively with time in other sedentary behaviors. To our knowledge, no studies have evaluated how Internet and computer use relate to other sedentary behaviors.

The aim of this study is to examine associations of Internet and computer use, specifically in leisure time (excluding occupational computer use), with overweight and obesity, leisure-time physical activity, and other sedentary behaviors, in a large socially-diverse sample of Australian adults.

## Methods

### Participants and Procedures

This study is part of an observational epidemiological study (PLACE: Physical Activity in Localities and Community Environments) conducted in urban areas of Adelaide, Australia during 2003 - 2004. Detailed methods of the study have been described elsewhere [23,24]. Briefly, a study sample was drawn from residential addresses within 32 neighbourhoods which are known to vary in socio-economic status. In each neighbourhood, 250 addresses were randomly selected and sent a letter of invitation to participate. Eligible respondents (English speaking, aged between 20 and 65, residing in private dwellings, and able to walk without assistance) who agreed to participate were mailed a survey that included questions about Internet and computer use, other sedentary behaviors, physical activity, body weight, height, and socio-demographic characteristics. Participant recruitment and data collection were handled in a series of waves, between July 2003 and June 2004, in order to obtain data from respondents across the range of seasons. A total of 2650 eligible participants returned the questionnaire. The return rate for those who completed the survey, as a proportion of those known to be contacted was 74.2%. The Behavioral and Social Sciences Ethics Committee of the University of Queensland approved the study.

### Measures

#### Leisure-Time Internet and Computer Use

Participants reported leisure-time Internet and computer use as part of a measurement tool assessing total leisure-time sedentary behavior in the last seven days. For each sedentary activity, the tool asks “How many days did you do this activity in the last 7 days”, followed by “On average, how many minutes did you do this activity on the days you did it”. This instrument has been shown to have acceptable reliability and validity, especially for Internet and computer use [25]. To evaluate the validity of this measure, three-day sedentary behavior logs were collected from 130 participants. The Spearman rank-order correlations showed that, compared with the three-day log, the last seven day recall measure was acceptable for Internet and computer use (*ρ* = 0.6). Test-retest reliability was evaluated in a sample of 145 participants. Intra-Class Correlations (ICC) indicated acceptable agreement for Internet and computer use (ICC = 0.62). The amount of leisure-time Internet and computer use was split into three categories: no, low (less than three hours per week), and high (three hours or more per week) Internet and computer use.

#### Overweight and Obesity

Body Mass Index (BMI; kg/m^2^) was calculated using self-reported height and weight and was categorized as either normal weight (< 25 kg/m^2^); overweight (≥ 25 and < 30 kg/m^2^) or obese (≥ 30 kg/m^2^). BMI was also used as a continuous variable.

Leisure-time physical activity was assessed using the long-form (31 items) International Physical Activity Questionnaire (IPAQ) [26]. Participants reported the number of days per week and the time spent per day on walking, as well as vigorous-intensity and moderate-intensity leisure-time activities, during the last seven days. The amount of leisure-time physical activity was split into three categories: low (less than one hour per week), medium (between one and three hours per week), and high (three or more hours per week) leisure-time physical activity.

#### Other Leisure-Time Sedentary Behaviors

The instrument applied to measure Internet and computer use [25] was also applied to measure the total of “other leisure-time sedentary behaviours [*sic*]”. This variable included time spent: reading, sitting when talking to friends or listening to music, talking on the phone, playing video games, watching television, and driving or riding in a car; it did not include Internet and computer use. The amount of other sedentary behaviors was split into: low (less than 2.5 hours per day), medium (between 2.5 and 5 hours per day), and high (5 or more hours per day).

### Statistical Analysis

One-way ANOVA and Chi-square tests were used for analysing differences in socio-demographic factors according to different categories of Internet and computer use. Multinomial logistic regression analyses were conducted to estimate associations of Internet and computer use with overweight and obesity (model 1), leisure-time physical activity (model 2), and other sedentary behaviors (model 3). The models were adjusted for age, gender, employment, level of education, overweight and obesity (only in models 2 and 3), other sedentary behaviors (only in models 1 and 2), and leisure-time physical activity (only in models 1 and 3). Binary logistic regression was conducted to estimate the odds ratios of being overweight or obese, comparing levels of Internet and computer use (no, low, and high Internet and computer use) and physical activity (low, medium, and high leisure-time physical activity). This model was adjusted for age, gender, education, employment, and other sedentary behaviors. Analyses were conducted using SPSS version 13.0. Significance was accepted at an alpha level of 0.05.

## Results

 Sample size was 2532 (1554 women, 978 men), after excluding missing values for Internet and computer use (n = 118). Average leisure-time Internet and computer use was 125.3 minutes per week (SD: 273.3). Table 1 shows socio-demographic characteristics for the total sample according to Internet and computer use. Participants with low Internet and computer use had the highest levels of educational attainment and employment, were younger, and participated in less other sedentary behaviors compared to participants with either high Internet and computer use or no use. Participants with high Internet and computer use had the highest BMI compared to the other groups and were more likely to be male.

**Table 1:** Sample characteristics for total group and according to computer and Internet use categories (mean ± SD or %)^a^

	Total Sample (N = 2650)	No Internet or computer use (N = 1093)	Low Internet and computer use (N = 983)	High Internet and computer use (N= 456)	*P*-value

Sex (% female)	64.0	68.7	65.4	50.1	< .001
Age (yr)	44.5 ± 12.3	45.8 ± 11.8	42.8 ± 12.4	44.1 ± 12.7	< .001
College or university degree (%)	46.3	36.9	55.8	51.0	< .001
Employed (%)	69.2	62.9	77.0	67.6	< .001
Leisure-time physical activity (hrs/week)	3.3 ± 4.5	3.1 ± 4.7	3.6 ± 4.5	3.2 ± 4.3	ns
Body Mass Index (kg/m^2^)	26.3 ± 6.4	25.9 ± 5.9	25.9 ± 5.6	27.5 ± 8.3	< .001
Other sedentary behaviours (hrs/week)	27.6 ± 16.9	27.5 ± 18.2	25.6 ± 14.3	32.2 ± 18.2	< .001

^a^Chi-squared and one-way ANOVA were used to examine differences between categories; ns is not significant.

### Overweight and Obesity by Internet and Computer Use

As shown in Table 2, leisure-time Internet and computer use was significantly associated with overweight and obesity. Compared to participants that reported no Internet and computer use, participants with low Internet and computer use were 1.3 times more likely to be overweight and 1.4 times more likely to be obese, and participants with high Internet and computer use were 1.5 times more likely to be overweight and 2.5 times more likely to be obese.

### Leisure-Time Physical Activity by Internet and Computer Use

Leisure-time physical activity was largely independent of leisure-time Internet and computer use. However, participants with low Internet and computer use were 1.3 times more likely to do more than three hours of leisure-time physical activity, when compared to non-users.

### Other Leisure-Time Sedentary Behaviors by Internet and Computer Use

Participants with low and high leisure-time Internet and computer use were respectively 1.8 and 2.5 times more likely to engage in more than five hours of other sedentary behaviors per day, when compared to participants that did not use the Internet and computer.

**Table 2:** Multinomial logistic regression models predicting overweight or obesity, leisure-time physical activity, and other sedentary behaviors by computer and Internet use^a^

			OR (95% CI)	OR (95% CI)

**Model 1: Weight status**		**Normal weight** **(N = 1187)**	**Overweight** **(N = 783)**	**Obese** **(N = 442)**
Internet and computer use				
	No use	Reference	1.00	1.00
	Low use	Category	1.30 (1.01 - 1.56)^b^	1.45 (1.10 - 1.92)^c^
	High use		1.46 (1.10 - 1.93)^c^	2.52 (1.81 - 3.51)^c^
**Model 2: Leisure-time Physical Activity (LTPA)**		**Low LTPA** **(N = 1375)**	**Medium LTPA** **(N = 721)**	**High LTPA** **(N = 429)**
Internet and computer use				
	No use	Reference	1.00	1.00
	Low use	Category	1.12 (0.87 - 1.44)	1.28 (1.02 - 1.60)^b^
	High use		0.81 (0.60 - 1.12)	0.83 (0.63 - 1.11)
**Model 3: Other Leisure-time Sedentary Behaviors (LTSB)**		**Low LTSB** **(N = 656)**	**Medium LTSB** **(N = 1045)**	**High LTSB** **(N = 557)**
Internet and computer use				
	No use	Reference	1.00	1.00
	Low use	Category	1.24 (0.99 - 1.55)	1.79 (1.30 - 2.46)^c^
	High use		0.99 (0.75 - 1.30)	2.50 (1.75 - 3.57)^c^

^a^Regression models were adjusted for gender, age, employment, educational attainment, other sedentary behaviors, leisure time physical activity, and BMI.

^b^ *P* < .05

^c^ *P* < .001

### Overweight and Obesity by Leisure-Time Physical Activity by Internet and Computer Use

**Figure 1:**
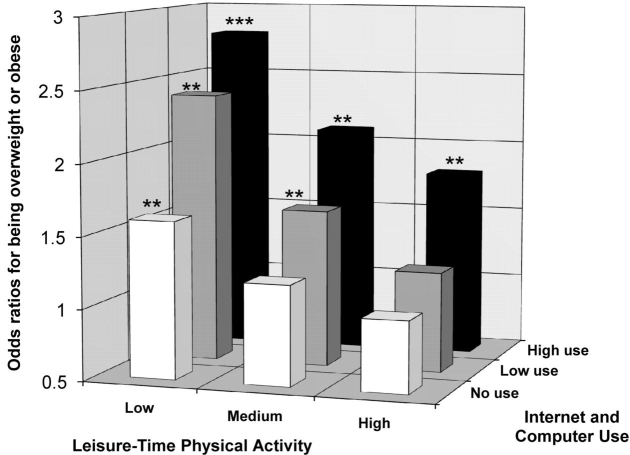
The odds ratios for being overweight or obese (BMI ≥ 25), according to combined categories of Internet and computer use (no, low, and high leisure-time Internet and computer use) and physical activity (low, medium, and high leisure-time physical activity). The reference category is having high leisure-time physical activity and not using the Internet and computer, for which the odds ratios are equal to 1. The significance levels on top of the figure bars are differences in relation to the reference category: ** P < .01; *** P < .001

Figure 1 shows the odds ratios for being overweight or obese were higher when leisure-time physical activity was lower and/or when Internet and computer use was higher. For example, participants with low leisure-time physical activity and high Internet and computer use were 2.77 times (95% CI = 1.86 - 4.12) more likely to be overweight or obese, and participants with high leisure-time physical activity and high Internet and computer use were 1.86 times (95% CI = 1.21 - 2.88) more likely to be overweight or obese, as compared to participants with high leisure-time physical activity and participants who did not use the Internet and computer.

## Discussion

The main finding of this study is that leisure-time Internet and computer use is strongly related to being overweight or obese, whereas it is largely independent of leisure-time physical activity. After adjusting for socio-demographic variables, leisure-time physical activity and other sedentary behaviors, participants who used the Internet and computer for three hours or more in the last seven days were 1.5 times more likely to be overweight and 2.5 times more likely to be obese compared to non-users. Although there are no direct comparisons with other studies for these outcomes in adults, they are in line with studies that report that higher amounts of time in sedentary behavior and television viewing are strongly associated with overweight and obesity [5-10,12,27].

The strong associations of leisure-time Internet and computer use with overweight and obesity may in part be explained by the association of leisure-time Internet and computer use with other leisure-time sedentary behaviors. Participants who had high Internet and computer use in their leisure time were 2.5 times more likely to engage in more than five hours of other sedentary behaviors per day. This is consistent with a study by Sugiyama et al [22] which showed that time spent watching TV was positively associated with other leisure-time sedentary behaviors; however, this was only the case for women.

Our results showed that leisure-time Internet and computer use was not strongly associated with leisure-time physical activity. Contrary to what might be expected, participants with low leisure-time Internet and computer use were slightly more likely to be in a higher leisure time physical activity category. While it is difficult to explain this outcome, it might be argued that it could be due to the higher socio-economic profile observed in participants with low Internet use. It is generally the case that those of higher socio-economic status are more physically active [28]. However, the analyses controlled for educational attainment and employment status, so such an interpretation would not apply to our findings. Other than this particular relationship, no associations between leisure-time Internet and computer use and physical activity were observed. This finding is, thus, for the major part consistent with studies that showed non-significant associations between Internet use and physical activity in children and adolescents [19-21] and those between TV viewing time and physical activity in adults [5,6,8-10,12]. From this perspective, the apparent paradox of increasing physical activity using an intervention delivery mode that promotes sedentary behavior (Internet and computer use) appears to be invalid. Our results suggest that the time that spent taking part in Internet interventions is not likely to displace leisure-time physical activity; hence, Internet interventions should be considered as an acceptable method to increase physical activity.

As may be seen in Figure 1, a combination of high Internet and computer use and low leisure-time physical activity was associated with a higher odds ratio of being overweight or obese. This finding is consistent with those of a study by Salmon et al [12] in which higher levels of TV viewing in combination with lower levels of physical activity participation were found to be associated with being overweight or obese. This figure also shows that adults who use the Internet and computer for more than three hours in their leisure time are significantly more likely to be overweight, even if they are highly active in their leisure time. Consistent with what was reported by Salmon et al [12], these findings suggest that, in order to reduce the prevalence of overweight and obesity, it may be important not only to increase participation in physical activity, but also to reduce time spent in sedentary behaviors, such as leisure-time Internet and computer use.

As the level of Internet penetration increases, its users become more representative of the general population; thus, gender, age, and socio-economic differences are diminishing [13]. Nevertheless, interesting socio-demographic profiles emerged when leisure-time Internet and computer use were categorized into different levels of usage. Our findings indicate that participants with low leisure-time Internet and computer use had the highest socio-economic profile, engaged in less time in other sedentary behaviors and were slightly more likely to do more leisure-time physical activity. On the other hand, participants with high leisure-time Internet and computer use had lower socio-economic profiles, engaged in more time in other sedentary behaviors, had a higher BMI, and were more likely to be male. This suggests that different levels of leisure-time Internet and computer use are related to different socio-demographic profiles and health behaviors.

Given the high prevalence of Internet use, and its potential impact on health, it is important to address health issues for Internet users. Internet interventions to reduce weight or increase physical activity are likely to be more effective if they take differences among Internet users into account. Although a substantial number of these Internet interventions have been implemented, no studies reported that participants were targeted differently based on their level of Internet and computer use [29,30]. Our findings suggest that doing so might be important. More efforts should be put in targeting participants with high Internet and computer use as compared to those with a low Internet and computer use (less than 3 hours a week). As indicated above, those with high Internet and computer use have an unfavorable health risk profile, and thus potentially may be more open to participating in such interventions, as they have higher levels of computer use in their leisure time.

The major limitations of this study are that it relies on self-reported measures and a cross-sectional design which does not allow determination of the causal direction of the results. More research, using objective measures and prospective study designs, is needed to evaluate these associations. A further limitation is that this study only investigated leisure-time behaviors, this prevents evaluating the impact of using the Internet and computer at work on physical activity and overweight and obesity. Nevertheless, the associations observed in this study indicate that the impact of Internet and computer use, when only used in leisure time, is strong enough to have an influence on health, and that this impact should be taken into consideration when developing new interventions targeting these leisure-time behaviors.

In summary, high levels of leisure-time Internet and computer use were associated with a higher BMI (even among those engaging in a high level of leisure-time physical activity) and higher levels of other leisure-time sedentary behaviors. However, Internet and computer use was mostly unrelated to leisure-time physical activity. These findings suggest that, in addition to nutritional and physical activity interventions, it may also be necessary to decrease time spent in sedentary behaviors (including leisure-time Internet and computer use) in order to reduce the risk of overweight and obesity. Furthermore, future Internet interventions to reduce weight or increase physical activity may need to differentiate between participants with different levels of leisure-time Internet and computer use, in order to increase their effectiveness. Our study is the first to evaluate these specific associations; hence, more research is needed to confirm these findings. More specifically, longitudinal studies are required to examine further the potential causal relationships between specific sedentary behaviors, such as Internet and computer use, and weight gain.

## Abbreviations

ANOVA: analysis of variance
BMI: body mass index
ICC: intra-class correlations
IPAC: International Physical Activity Questionnaire
PLACE: Physical Activity in Localities and Community Environments
